# PyBioMed: a python library for various molecular representations of chemicals, proteins and DNAs and their interactions

**DOI:** 10.1186/s13321-018-0270-2

**Published:** 2018-03-20

**Authors:** Jie Dong, Zhi-Jiang Yao, Lin Zhang, Feijun Luo, Qinlu Lin, Ai-Ping Lu, Alex F. Chen, Dong-Sheng Cao

**Affiliations:** 1 Xiangya School of Pharmaceutical Sciences, Central South University, No. 172, Tongzipo Road, Yuelu District, Changsha, People’s Republic of China; 2 College of Food Science and Engineering, National Engineering Laboratory for Deep Processing of Rice and Byproducts, Central South University of Forestry and Technology, Changsha, China; 3 Institute for Advancing Translational Medicine in Bone and Joint Diseases, School of Chinese Medicine, Hong Kong Baptist University, Hong Kong SAR, China; 4 Center for Vascular Disease and Translational Medicine, Third Xiangya Hospital, Central South University, Changsha, People’s Republic of China

**Keywords:** Molecular representation, Molecular descriptors, Python library, Chemoinformatics, Data integration, Bioinformatics

## Abstract

**Background:**

With the increasing development of biotechnology and informatics technology, publicly available data in chemistry and biology are undergoing explosive growth. Such wealthy information in these data needs to be extracted and transformed to useful knowledge by various data mining methods. Considering the amazing rate at which data are accumulated in chemistry and biology fields, new tools that process and interpret large and complex interaction data are increasingly important. So far, there are no suitable toolkits that can effectively link the chemical and biological space in view of molecular representation. To further explore these complex data, an integrated toolkit for various molecular representation is urgently needed which could be easily integrated with data mining algorithms to start a full data analysis pipeline.

**Results:**

Herein, the python library *PyBioMed* is presented, which comprises functionalities for online download for various molecular objects by providing different IDs, the pretreatment of molecular structures, the computation of various molecular descriptors for chemicals, proteins, DNAs and their interactions. *PyBioMed* is a feature-rich and highly customized python library used for the characterization of various complex chemical and biological molecules and interaction samples. The current version of *PyBioMed* could calculate 775 chemical descriptors and 19 kinds of chemical fingerprints, 9920 protein descriptors based on protein sequences, more than 6000 DNA descriptors from nucleotide sequences, and interaction descriptors from pairwise samples using three different combining strategies. Several examples and five real-life applications were provided to clearly guide the users how to use *PyBioMed* as an integral part of data analysis projects. By using *PyBioMed*, users are able to start a full pipelining from getting molecular data, pretreating molecules, molecular representation to constructing machine learning models conveniently.

**Conclusion:**

*PyBioMed* provides various user-friendly and highly customized APIs to calculate various features of biological molecules and complex interaction samples conveniently, which aims at building integrated analysis pipelines from data acquisition, data checking, and descriptor calculation to modeling. *PyBioMed* is freely available at http://projects.scbdd.com/pybiomed.html.
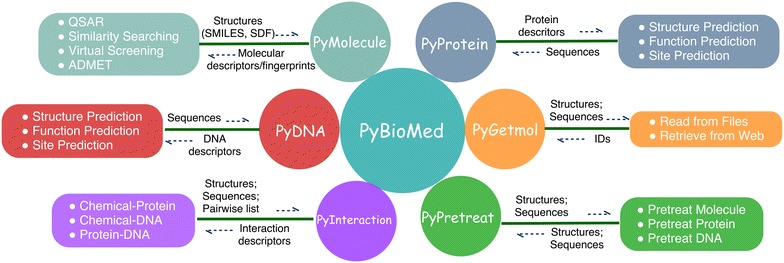

## Background

With the increasing development of biotechnology and informatics technology, the past decade has seen an exceptional growth in publicly available data in chemistry and biology, especially in human-specific molecular interaction data. The heterogeneity of data in databases poses a significant challenge to their integration and analysis in practice [1, 2]. However, the bioinformatics and the cheminformatics communities have evolved more or less independently, e.g., with an emphasis on macro biomolecules and chemical compounds, respectively. Investigation of interactions is a complex molecular recognition process, which is not only related to the bioinformatics projects that aim at a systematic analysis of the structure and function of proteins and DNAs that scales to the genome level, but also to the chemoinformatics projects that are devoted to the analysis of structure and biological activity of chemicals. More importantly, systematic investigation of generated knowledge in both the chemical and biological knowledge spaces is required, especially in the scenarios of identifying both new targets and their potential ligands, discovering potential biomarkers for complex diseases, understanding the mechanism of interactions, and discovering new regulatory mechanism etc. [3–8]. Therefore, it is very necessary to build informatics platforms for unified data or knowledge representation that can integrate the existing efforts from both communities.

Furthermore, wealthy information in these data needs to be extracted and then transformed to useful knowledge by various data mining and artificial intelligent methods. Lots of machine learning methods have been elaborately developed to mine useful biomedicine information [9–16]. However, in order to apply various machine learning approaches on molecular data, it is a common practice to encode molecular information as numerical features. The type of encoding, however, can significantly affect analyses, and choosing a precise and effective encoding is a critical step. Molecular descriptors are one of the most powerful approaches to characterize the biological, physical, and chemical properties of molecules and have long been used in various studies for understanding molecular interactions or drug discovery. These descriptors capture and magnify distinct aspects of molecular topology in order to investigate how molecular structure affects molecular property. Molecular features have frequently been used in the development of machine learning in QSAR/QSPR [17, 18], virtual screening [19], similarity search [20], drug absorption, distribution, metabolism, elimination and toxicity (ADMET) eavaluation [21–24], protein structural and functional classes [25, 26], protein–protein interactions [27], compound–protein interactions [28–31], subcellular locations and peptides of specific properties [32], meiotic recombination hot spots [33], nucleosome positioning in genomes and other drug discovery processes [34]. In terms of molecular representation importance, some web servers and stand-alone programs, such as *RDKit* [35], *CDK* [36], *rcdk*, *PaDEL* [37], *Cinfony* [38], *Chemopy* [39], *ChemDes* [40], *BioJava* [41], *BioTriangle* [42], *bioclipse* [43], *propy* [44], *PyDPI* [45], *Biopython*, *repDNA* [46], *CDK*-*Taverna* [47], *protr/protrWeb* [48], *ChemmineR* [49], and *Rcpi* [50] have been established to calculate such descriptors. However, currently available solutions are often limited to certain types of descriptors, lack flexibility, and usually difficult to seamlessly integrate into the predictive modeling pipeline. We still urgently need a comprehensive and flexible toolkit to integrate these separate functionalities into a uniform framework, and further enable us to build a full analysis pipeline.

Here, we developed *PyBioMed*, a python library, for realizing comprehensive molecular representation of various molecular objects and basic data analysis tasks. *PyBioMed* mainly focuses on the study of molecular representation techniques for not only single molecular objects, but also interactions between any two individual molecules from pairwise samples. To easily use the *PyBioMed* utilities and functionalities, we provide a uniform interface and highly customized modules in the library to perform data analysis. To introduce and describe *PyBioMed* utility and application, we selected five wide applications as examples to show that *PyBioMed* can be used as an integral part of an analytical pipeline. Our computational algorithms are extensively tested and the computed features have also been used in a number of published studies. We recommend *PyBioMed* to analyze and represent various complex molecular data under investigation. Further, we hope that the library will be incorporated to other research projects such as data analysis pipeline, web server and software applications, in which the molecular representation will play an important role.

## Implementation

The library *PyBioMed* is implemented in python. It is mainly based on the *RDKit* and *pybel* implementation, licensed under BSD 3-Clause License, and depends on the following python modules: *scipy*, *numpy*, *os* and *sys*. The library is mainly divided into six highly customized modules, namely: (1) *PyGetMol*, (2) *PyPretreat*, (3) *PyMolecule*, (4) *PyProtein*, (5) *PyDNA* and (6) *PyInteraction*. The modules allow the users to get various molecular objects by different ways, pretreat these molecular objects, and finally calculate chemical descriptors, protein descriptors, DNA descriptors and interaction descriptors by different functionalities. The implementation of these six modules is independent, and each module could perform the specific function. Additionally, four additional files include examples and applications, test modules and help documentations, which will greatly facilitate the use of the library. The *PyBioMed* library is freely available from the home page of the *PyBioMed* project. URL: http://projects.scbdd.com/pybiomed.html. The formatted documentation of the library is designed based on the sphinx language. The documentation of 149 pages includes the introduction of the library, the installation of the library, how to use each function or module by an example, five real-life applications widely covering different study fields, the detailed documentation of *PyBioMed* APIs, how to test the library, and the download links for detailed descriptor introduction. The users could learn all knowledge of the library by looking up the detailed documentation. The main functionalities of the library are presented in the following subsections.

### Downloading and reading molecular files

Before various studies, it is the first step for the researchers to conveniently obtain various molecular objects from various web sources such as chemical structures, protein sequences, and DNA sequences. The *PyBioMed* library designed a specific module called *PyGetMol* to realize the aim. The *PyGetMol* module is responsible for obtaining and reading various molecular objects, and it contains three functions for downloading molecular objects (*Getmol*, *GetProtein*, *GetDNA*) and various functions for reading molecular objects. The *Getmol* module provides multiple tools to get molecular structures by the molecular ID from websites including NCBI, EBI, CAS, KEGG and DrugBank. The *GetProtein* module provides the tool to get protein sequence by the PDB ID and UniProt ID. The *GetDNA* module provides the tool to get DNA sequence by the Gene ID from websites. Additionally, they are also responsible for reading various molecular objects in different formats, such as SDF, MOL, InChI and SMILES for chemicals, FASTA for proteins and DNAs. The incorporation of these functionalities makes *PyBioMed* easily accessible in various cheminformatics applications.

### Pretreating molecular objects

The check and preprocessing for various molecular objects is of high importance for subsequent descriptor calculation and data analysis, especially those molecular objects from web sources. Some molecules may contain structure defects to a certain extent, and therefore seriously influence or even destroy the subsequent descriptor calculation. The *PyPretreat* module in the library provides three specific functions which are responsible for pretreating three different molecular objects. The user could check the protein and DNA sequences by *PyPretreatPro* and *PyPretreatDNA*, respectively. The two functions mainly check whether there are additional amino acids types or DNA basic group types or not. If the sequence is right, the result goes back to the number of amino acids and nucleotides or not, otherwise, the result is 0. For chemical compounds, the pretreating step seems to be complex. *PyPretreatMol* pretreats a chemical structure in the following ways: (1) normalization of functional groups to a consistent format; (2) recombination of separated charges; (3) breaking of bonds to metal atoms; (4) competitive deionization to ensure strongest acids ionize first in partially ionize molecules; (5) tautomer enumeration and canonicalization; (6) neutralization of charges; (7) standardization or removal of stereochemistry information; (8) filtering of salt and solvent fragments; (9) generation of fragment, isotope, charge, tautomer or stereochemistry insensitive parent structures; (10) validations to identify molecules with unusual and potentially troublesome characteristics. The functionalities can be highly customized by setting corresponding parameters according to the job demand. Alternatively, the users could also pretreat the molecular structure using all functions by *StandardSmi* function.

### Calculating descriptors form chemicals, proteins and DNAs

Molecular representation is the core feature of the *PyBioMed* library. ‘The molecular descriptor is the final result of a logic and mathematical procedure which transforms chemical or biological information encoded within a symbolic representation of a molecule into a useful number or the result of some standardized experiment’ [51]. Molecular representation plays a fundamental role in chemoinforamtics and bioinformatics, and lies at the heart of ligand-based drug design. It is the first even the most key step in the data analysis tasks. A large number of molecular descriptors from chemicals, proteins and DNAs could be easily calculated by the *PyBioMed* library, which covers wide applications in various cheminformatics and bioinformatics projects.

### Molecular descriptors based on chemical structures

The *PyMolecule* module in *PyBioMed* is responsible for calculating the commonly used structural and physicochemical descriptors. It computes twelve feature groups composed of fourteen descriptors that include 775 descriptor values. These descriptors capture and magnify distinct aspects of chemical structures, including constitutional descriptors, topological descriptors, kappa shape indices, connectivity indices, Burden descriptors, E-state indices, charge descriptors, Basak information indices, autocorrelation descriptors, molecular properties, MOE-type descriptors, and pharmacophore descriptors. A detailed list of descriptors for chemicals covered by *PyBioMed* is summarized in Table 1. The usefulness of molecular descriptors in the representation of molecular information is reflected in their widespread adoption and use across a broad range of applications and methodologies, as reported in a large number of published articles [18, 22, 52, 53]. More detailed description and references can be found in the documentation of *PyBioMed*. We could import the corresponding module to calculate the molecular descriptors as need. Alternatively, an easier way to compute these descriptors is construct a *PyMolecule* object, which encapsulates all methods for the calculation of descriptors.

**Table 1:** Molecular descriptors of chemicals calculated by *PyBioMed*

Feature group	Features	Number of descriptors
Constitution	Molecular constitutional descriptors	30
Topology	Topological descriptors	35
Connectivity	Molecular connectivity indices	44
E-state	E-state descriptors	237
Bask	Bask descriptors	21
Burden	Burden descriptors	64
Kappa	Kappa shape descriptors	7
Autocorrelation	Moreau–Broto autocorrelation	32
	Moran autocorrelation	32
	Geary autocorrelation	32
Charge	Charge descriptors	25
Property	Molecular property	6
MOE-type	MOE-type descriptors	60
Pharmacophore	Chemically advanced template search (CATS)	150

Besides molecular descriptors, *PyBioMed* also realizes the computation of a number of molecular fingerprints, and a specific *fingerprint* module is designed to achieve the aim. Molecular fingerprints are string representations of chemical structures, which consist of bins, each bin being a substructure descriptor associated with a specific molecular feature. 19 types of molecular fingerprints and substructure fragments are provided in *PyBioMed*, including topological fingerprints, E-state fingerprints, MACCS keys, FP4 keys, atom pairs fingerprints, PubChem fingerprints, topological torsion fingerprints, and Morgan/circular fingerprints etc. (see Table 2). The usefulness of these molecular fingerprints covered by *PyBioMed* have been sufficiently demonstrated by a number of published studies of the development of machine learning classification systems in QSAR/SAR, drug ADME/T prediction, similarity searching, clustering, ranking and classification [54].

**Table 2:** Molecular fingerprints of chemicals calculated by *PyBioMed*

Feature group	Features	Number of descriptors
Substructure-based fingerprints	MACCS fingerprints	166
	E-state fingerprints	79
	Ghose–Crippen fingerprints	110
	FP3 fingerprints	210
	FP4 fingerprints	307
	PubChem fingerprints	881
Fingerprints	Daylight-type fingerprints	2048
	Atom pairs fingerprints	2048
	Topological torsion fingerprints	2048
	FP2 fingerprints	1024
	ECFP2 fingerprints	1024
	ECFP4 fingerprints	1024
	ECFP6 fingerprints	1024
	FCFP2 fingerprints	1024
	FCFP4 fingerprints	1024
	FCFP6 fingerprints	1024
	Morgan fingerprints	1024
	Pharm2D2point fingerprints	135
	Pharm2D3point fingerprints	2135

### Protein or peptide descriptors based on amino acid sequences

The *PyProtein* module in *PyBioMed* is responsible for calculating the widely used structural and physicochemical features of proteins and peptides from amino acid sequences. It computes five feature groups composed of fourteen features, including amino acid composition, dipeptide composition, tripeptide composition, normalized Moreau–Broto autocorrelation, Moran autocorrelation, Geary autocorrelation, sequence-order-coupling number, quasi-sequence-order descriptors, composition, transition and distribution of various structural and physicochemical properties, and two types of pseudo amino acid composition (PseAAC) descriptors. These features could be generally regarded as different Chou’s PseAAC modes. In addition, it can also easily compute previous descriptors based on user-defined properties, which are automatically available from the AAindex database. A list of features for proteins and peptides covered by *PyBioMed* is summarized in Table 3. These features have been used for predicting protein- and peptide-related problems by using machine learning methods. More detailed description and references can be found in the documentation of *PyBioMed*.

**Table 3:** Protein descriptors of proteins or peptides calculated by *PyBioMed*

Feature group	Features	Number of descriptors
Amino acid composition	Amino acid composition	20
	Dipeptide composition	400
	Tripeptide composition	8000
Autocorrelation	Normalized Moreau–Broto autocorrelation	240^a^
	Moran autocorrelation	240^a^
	Geary autocorrelation	240^a^
CTD	Composition	21
	Transition	21
	Distribution	105
Conjoint triad	Conjoint triad features	343
Quasi-sequence order	Sequence order coupling number	60
	Quasi-sequence order descriptors	100
Pseudo amino acid composition	Pseudo amino acid composition	50^b^
	Amphiphilic pseudo amino acid composition	50^c^

^a^The number depends on the choice of the number of properties of amino acid and the choice of the maximum values of the *lag*. The default is use eight types of properties and *lag* = 30

^b^The number depends on the choice of the number of the set of amino acid properties and the choice of the *λ* value. The default is use three types of properties proposed by Chou et al. and *λ* = 30

^c^The number depends on the choice of the *λ* value. The default is that *λ *= 15

In fact, the abovementioned features can be regarded as different Chou’s PseAAC modes [55]. For example, amino acid, dipeptide, tripeptide, or *k*-mer peptide (*k *= 4, 5,…) compositions are just different modes of Chou’s PseAAC. Moreover, the higher-level features such as GO (Gene Ontology) information, FunD (Functional Domain) information, and Sequential Evolution information are also skillfully fused into the Chou’s PseAAC descriptors to characterize different protein information which is widely used for solving various biological problems. An excellent review by Chou has pointed out their relevancy.

### DNA descriptors based on nucleotide sequences

The *PyDNA* module in *PyBioMed* is responsible for calculating the widely used structural and physicochemical features of DNAs from nucleotide sequences. Generally, three groups of features from nucleotide sequences are calculated to represent DNA in *PyBioMed*. (1) three nucleic acid composition features describing the local sequence information by means of *k*-mers (subsequences of DNA sequences); (2) six autocorrelation features describing the level of correlation between two oligonucleotides along a DNA sequence in terms of their specific physicochemical properties; (3) six pseudo nucleotide composition features, which can be used to represent a DNA sequence with a discrete model or vector yet still keep considerable sequence order information, particularly the global or long-range sequence order information, via the physicochemical properties of its constituent oligonucleotides. A detailed list of descriptors for DNAs covered by *PyBioMed* is summarized in Table 4. The usefulness of these features covered by *PyBioMed* for representing DNA sequence information have been sufficiently demonstrated by a number of published studies in computational genomics and genome sequence analysis. More detailed description and references can be found in the documentation of the *PyBioMed* library. There are two ways to calculate DNA descriptors in the *PyDNA* module. One is to directly use the corresponding methods, the other one is firstly to construct a *PyDNA* class and then run their methods to obtain the descriptors. The users could select one or more groups to represent DNAs under investigation. It should be noted that the output is a dictionary form, whose keys and values represent the descriptor name and the descriptor value, respectively. The user could clearly understand the meaning of each descriptor.

**Table 4:** DNA descriptors of DNAs calculated by *PyBioMed*

Feature group	Features	Number of descriptors^a^
Nucleic acid composition	Basic kmer	16
	Reverse compliment kmer	12
Autocorrelation	Dinucleotide-based auto covariance	76
	Dinucleotide-based cross covariance	2812
	Dinucleotide-based auto-cross covariance	2888
	Trinucleotide-based auto covariance	24
	Trinucleotide-based cross covariance	264
	Trinucleotide-based auto-cross covariance	288
Pseudo nucleic acid composition	Pseudo dinucleotide composition	18
	Pseudo k-tuple nucleotide composition	18
	Parallel correlation pseudo dinucleotide composition	18
	Parallel correlation pseudo trinucleotide composition	66
	Series correlation pseudo dinucleotide composition	90
	Series correlation pseudo trinucleotide composition	88

^a^The number depends on the choice of the values of the parameters in the formula. Here, the number of each type of descriptors is based on the default parameter value. For detailed information, please refer to the documentation section in the *PyBioMed* manual

### Interaction descriptors based on pairwise samples

#### Descriptors from the interaction between two molecules with the same type

The interaction between two molecules with the same type includes drug–drug interaction, protein–protein interaction, etc. However, the construction process of the interaction descriptors of them is similar to each other. We will show how to construct an interaction feature by the protein–protein interaction example. Let **F**_**a**_ = {**F**_**a**_(i), i = 1, 2,…, p} and **F**_**b**_ = {**F**_**b**_(i), i = 1, 2,…, p} are the two descriptor vectors for interaction protein A and protein B, respectively. There are three methods to construct the interaction descriptor vector **F** for A and B:

1. Two vectors **F**_**ab**_ and **F**_**ba**_ with dimension of 2*p* are constructed: **F**_**ab**_ = (**F**_**a**_, **F**_**b**_) for interaction between protein A and protein B and **F**_**b**a_ = (**F**_**b**_, **F**_**a**_) for interaction between protein B and protein A.
2. One vector **F** with dimension of 2*p* is constructed: **F** = {**F**_**a**_(i) + **F**_**b**_(i), **F**_**a**_(i) × **F**_**b**_(i), i = 1, 2,…, p}.
3. One vector **F** with dimension of *p*^2^ is constructed by the tensor product: **F** = {**F**(k) = **F**_**a**_(i) × **F**_**b**_(j), i = 1, 2,…, p, j = 1, 2,…, p, k = (i − 1) × p + j}.

#### Descriptors from the interaction between two molecules with different types

The interaction between the molecules with different types includes chemical–protein interaction, protein–DNA interaction, and chemical–DNA interaction. However, the calculation of these interaction descriptors is similar to each other. Likewise, we will show how to construct an interaction feature by the chemical–protein interaction example. There are two methods for construction of descriptor vector **F** for chemical–protein interaction from the protein descriptor vector **F**_**t**_ (**F**_**t**_(i), i = 1, 2,…, p_t_) and chemical descriptor vector **F**_**d**_ (**F**_**d**_(i), i = 1, 2,…, p_d_):

1. One vector **V** with dimension of p_t_ + p_d_ is constructed: **F** = (**F**_**t**_, **F**_**d**_) for interaction between protein t and chemical d.
2. One vector **V** with dimension of p_t_ × p_d_ is constructed by the tensor product: **F** = {**F**(k) = **F**_**t**_(i) × **F**_**d**_(j), i = 1, 2,…, p_t_, j = 1, 2,…, p_d_, k = (i − 1) × p_t_ + j}.

### How to use *PyBioMed* functions

In order to familiarize the users with *PyBioMed*, It is recommended that the user works through the tutorial examples provided. The tutorial will go through the process of installing and running an example in some detail. The *PyBioMed* library would be applied to solve various research tasks in the field of cheminformatics, bioinformatics and systems biology. We introduced five examples of its applications in the documentation including Caco-2 cell permeability, aqueous solubility, drug–target interaction data, protein subcellular location, and nucleosome positioning in genomes. Next, we will briefly introduce the installation of *PyBioMed*, and how to calculate molecular descriptors by writing few lines of codes.

*PyBioMed* has been successfully tested on Linux and Windows systems. The installation process of *PyBioMed* is very easy. However, the user first needs to install *RDKit* and *pybel* successfully. The detailed installation steps are described in the README page of the GitHub repository of *PyBioMed.*

There are two means to compute these molecular descriptors from small molecules. One is to use the built-in modules. We could import related functions to compute these features. For example, the topology module includes a number of functionalities used for calculating various topological descriptors. The user could conveniently use them as need.
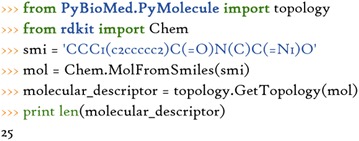


The other is to call the related class by importing the class module, which encapsulates commonly used descriptor calculation methods. *Pymolecule*, *Pyprotein* and *Pydna* are responsible for the calculation of chemical descriptors, protein descriptors, and DNA descriptors, respectively. We could construct a corresponding object with a molecule input, and then call corresponding methods to calculate these features.
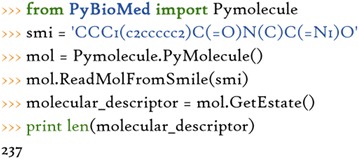


The interaction descriptors for pairwise samples are calculated as follows:
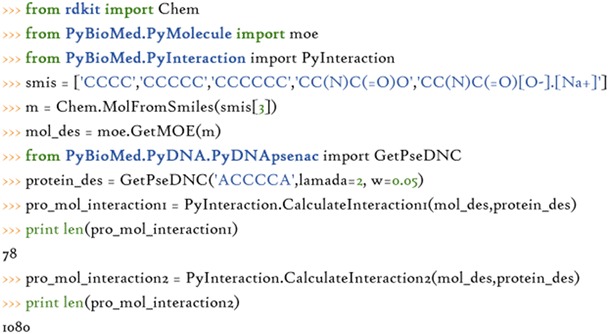


## Discussion

**Fig. 1:**
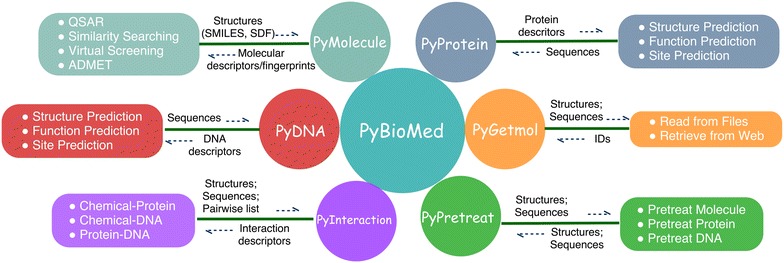
The main modules of the *PyBioMed* library and their corresponding wide applications in chemoinformatics, bioinformatics and drug discovery process

Considering the amazing rate at which data are accumulated in chemistry and biology fields, new tools that process and interpret large and complex interaction data are increasingly important. However, to our knowledge, no open source or freely available tool exists to perform all these functions above in a uniform framework. *PyBioMed* is a powerful python library for the extraction of features of complex interaction data. After representation, different statistical learning tools can be applied for further analysis and visualization of the data. Several case studies from wide applications show how *PyBioMed* was used to describe various molecular features and establish a model in a routing way (See documentation). The application domain of *PyBioMed* is not limited to the specific data type. It can, as Fig. 1 shows, be applied to a broad range of scientific fields such as QSAR/SAR, similarity search, virtual screening, ADMET prediction, ligand-based drug discovery, protein function/substructure/family classification, subcellular locations, post-translational modification (PTM), DNA structure/function/site prediction, and various interaction data analysis such as drug-target/drug interaction and protein–protein interaction studies. We expect that *PyBioMed* will better assist chemists, pharmacologists and biologists in characterizing, analyzing, and comparing complex molecular objects.

As mentioned in the background, there are several tools that have been developed to calculate chemical, protein or DNA/RNA description. However, *PyBioMed* is unique because it can be used to extract features of complex interaction data. Commonly, these tools can be divided into three types: software production, software package and web server. Software production tends to be commercial; web server tries to provide user-friendly way to beginners or users without programming skills; software package provides most flexible way to calculate descriptors in different application scenarios. The three types of tools have their own advantages. Here, *PyBioMed* belongs to software package of Python community. In order to give a more clear insight into the different software packages, we made a comparison between them (see Table 5).

**Table 5:** Differences of software packages for descriptor calculation

Tool names	Retrieving molecules	Pretreating molecules	Chemical descriptors/fingerprints	Protein descriptors	DNA/RNA descriptors	Interaction descriptors
PyBioMed	√	√	√	√	√	√
PyDPI			√	√		√
ChemoPy			√			
Cinfony			√			
RDKit		√	√			
CDK			√			
rcdk			√			
PaDEL			√			
BioJava	√	√		√		
Rcpi			√	√		√
ChemmineR	√		√			
Propy				√		
RepDNA					√	

From the Table 5, we can see: (1) most of these tools can only realize one or two functionalities while *PyBioMed* made all these functionalities into a uniform framework. (2) For some tools, the implications are actually not the same though the same functionality is ticked. Both *RDKit* and *PyBioMed* have the function of ‘Pretreating molecules’, while *PyBioMed* reorganized and wrapped the basic functions of *RDKit* into 10 new functions. By combining these functions in different orders and setting the corresponding parameters, users can realize the customized pretreating process. The specific functions for pretreating molecules and calculating protein descriptors of *BioJava* and *PyBioMed* are completely different. *ChemminR* can retrieve molecules from PubChem database while *PyBioMed* can retrieve molecules from NCBI, CAS, KEGG databases. *ChemminR* can calculate some basic molecular properties while *PyBioMed* can calculate various and diverse molecular descriptors, especially a series of featured fingerprints. *PyDPI* is able to calculate interaction descriptors but only limited to chemical–protein interaction feature. *PyBioMed* realized to represent nine types of interaction features. (3) The application scenarios are not the same though some tools can calculate the same kinds of descriptors. For example, *BioJava* is for Java environment, and *ChemminR* is for R environment, while *PyBioMed* is designed for Python environment. In summary, PyBioMed is a different tool from them and provides lots of unique functionalities, especially when extracting features of complex interaction data and realizing a full data analysis pipeline.

## Conclusion

*PyBioMed* provides a freely available and ease-to-use python library to calculate various features of biological molecules and complex interaction samples conveniently. It makes a step in this direction providing a way to fully integrate information from chemical space and biology space into an interaction space, which cannot be performed by other existing tools. As far as we know, *PyBioMed* is the first python library that calculates both individual descriptors of three types of molecular objects and any interactions between two of them in a uniform framework. It provides not only the detailed information about all descriptors and how to calculate them but also several tutorials and corresponding model scripts for different applications. In addition, the functions and modules related in *PyBioMed* and the stability of the library was extensively tested. We hope that the library will be helpful when exploring questions concerning structures, functions and interactions of various molecular data in the context of chemoinformatics. We also expect that our/other groups may use the free code of *PyBioMed* and the new machine learning models to implement public web servers. The increasingly diversified and further applications of molecular descriptors urge new descriptors and new tools to be developed, and researches have achieved some results [56–59]. In future work, we plan to apply the integrated features on various biological research questions, and to extend the range of functions with new promising descriptors for the coming versions of the library.
